# The membrane-associated β2e-subunit of voltage-gated calcium channels translocates to the nucleus and regulates gene expression

**DOI:** 10.3389/fphys.2025.1555934

**Published:** 2025-04-14

**Authors:** Erick Miranda-Laferte, Katalin Barkovits, Svitlana Rozanova, Nadine Jordan, Katrin Marcus, Patricia Hidalgo

**Affiliations:** ^1^ Institute of Biological Information Processing (IBI-1)- Molecular and Cellular Physiology, Forschungszentrum Jülich, Jülich, Germany; ^2^ Medizinisches Proteom-Center, Medical Faculty, Ruhr-University Bochum, Bochum, Germany; ^3^ Medical Proteome Analysis, Center for Protein Diagnostics (PRODI), Ruhr-University Bochum, Bochum, Germany; ^4^ Institute of Biochemistry, Heinrich-Heine University, Düsseldorf, Germany

**Keywords:** voltage-gated calcium channels, CaVβ, nuclear translocation, nuclear localization signal, PLC, cellular signaling, β-subunit voltage-gated calcium channel, Phospholipase C

## Abstract

The β-subunit (Cavβ) is a central component of the voltage-gated calcium channel complex. It lacks transmembrane domains and exhibits both channel-related and non-related functions. Previous studies have shown that, in the absence of the Cavα1 pore-forming subunit, electrostatic interactions between the N-terminus of Cavβ2e and the plasma membrane mediate its anchoring to the cell surface. Here, we demonstrate that, upon phospholipase C activation, Cavβ2e dissociates from the plasma membrane and homogeneously distributes between the cytosol and the nucleus. Mutagenesis analysis identified critical residues in the N-terminus of the protein, including a stretch of positively charged amino acids and a dileucine motif, which serve as nuclear import and export signals, respectively. Fusion of the Cavβ2e N-terminus to a trimeric YFP chimeric construct shows that this segment suffices for nuclear shuttling. Thus, the N-terminus of Cavβ2e emerges as a regulatory hotspot region controlling the subcellular localization of the protein. Quantitative mass spectrometry analysis revealed that the heterologous expression of a nuclear-enriched Cavβ2e mutant regulates gene expression. Our findings demonstrate the presence of active nuclear localization signals in Cavβ2e that enables its nuclear targeting and regulation of protein expression. Furthermore, they establish the membrane-associated Cavβ2e as a novel signaling mediator within the phospholipase C cascade.

## Introduction

Voltage-gated calcium channels (VGCCs) are heteromultimeric proteins composed of a pore-forming Cavα1 subunit and accessory subunits Cavβ, Cavα2δ, and Cavγ (Hofmann et al., 2014). VGCCs regulate calcium influx into cells following plasma membrane depolarization, playing critical roles in processes such as action potential duration, excitation-contraction coupling, and gene expression (Buraei and Yang, 2010). The Cavβ subunit is a member of the membrane-associated guanylate kinase (MAGUK) family, known for its scaffolding properties and its interaction with Cavα1 to regulate VGCC membrane trafficking and calcium influx (Buraei and Yang, 2013). Apart from its role on VGCCs regulation, Cavβ has been implicated in various non-channel-related functions (Belkacemi et al., 2018; Conrad et al., 2021; Cruz-Garcia et al., 2021; Gonzalez-Gutierrez et al., 2007; Hidalgo and Neely, 2007; Hofmann et al., 2014; Hofmann et al., 2015; Miranda-Laferte et al., 2011; Pickel et al., 2021).

The Cavβ2 isoform is predominantly expressed in the heart (Meissner et al., 2011) but is also found in the brain, aorta, and T cells (Buraei and Yang, 2010). Cavβ2 exists in five splice variants (β2a-β2e), differing in the first amino acids of the N-terminal variable (NTv) region, which influence the electrophysiological properties when coexpressed with Cavα1 (Chien et al., 1998; Link et al., 2009; Miranda-Laferte et al., 2014; Takahashi et al., 2003). Notably, Cavβ2a and Cavβ2e can associate with the plasma membrane independently of Cavα1, with Cavβ2e binding to the membrane through electrostatic interactions between its positively charged N-terminal stretch and the negatively charged phospholipids of the lipid bilayer (Kim et al., 2015a; Miranda-Laferte et al., 2014).

While studies have demonstrated that several non-membrane-associated Cavβ isoforms translocate to the nucleus to regulate gene expression (Corzo-López et al., 2023; Etemad et al., 2014; Hibino et al., 2003; Pickel et al., 2021; Subramanyam et al., 2009; Taylor et al., 2014; Vergnol et al., 2022; Zhang et al., 2010), the nuclear translocation of a membrane-associated Cavβ2 isoform and its potential involvement in signaling and gene regulation pathways remains unexplored.

Phospholipase C (PLC) activation, triggered by various G-protein-coupled receptors (GPCRs), plays a central role in cellular signaling by hydrolyzing phosphatidylinositol 4,5-bisphosphate (PIP2) into the second messengers inositol 1,4,5-trisphosphate (IP3) and diacylglycerol. IP3 releases Ca^2+^ from the endoplasmic reticulum, leading to diverse cellular outcomes, including changes in protein localization (Nakamura and Fukami, 2017). Previous studies have demonstrated that PLC signaling, induced by M1 muscarinic GPCR activation, promotes the release of Cavβ2e from the plasma membrane (Kim et al., 2015b).

In this study, we investigate the cellular localization of Cavβ2e following PLC activation and explore the underlying molecular mechanisms and potential functional consequences of this protein relocalization.

## Materials and methods

### cDNA constructs

The rat Cavβ2e cDNA (accession number: Q8VGC3-4) was cloned into a pcDNA3.1 YFP vector, resulting in the generation of the pcDNA3.1 Cavβ2e-YFP vector. Cavβ2e mutants, which were used for transient cell transfections, were created using standard overlapping PCR techniques with the pcDNA3.1 Cavβ2e-YFP vector as a template. The PCR fragments containing the Cavβ2e mutations were then subcloned into the pcDNA3.1 Cavβ2e-YFP vector using conventional molecular biology methods.

To obtain the wild-type Cavβ2e-NTv-YFP_3_ construct and its mutant variants, PCRs were conducted using the pcDNA3.1-YFP_3_ vector as a template. This vector contained three tandems, in-frame, repeats of YFP. The forward primers employed for each PCR started at the ATG of the first YFP in the vector and incorporated, at the 5′end as a hanging sequence, the sequences corresponding to the wild-type or mutant Cavβ2e-NTv fragments (amino acids 1–23). The reverse primer annealed at the C-terminus of the first YFP in the vector. Subsequently, the resulting PCR fragments were cloned into the aforementioned pcDNA3.1-YFP_3_ vector using conventional molecular biology methods.

The pcDNA5TM/FRT/TO Cavβ2e-mRFP and pcDNA5TM/FRT/TO Cavβ2e-L9A-mRFP constructs were generated for the establishment of the Flp-In™ T-REx™ 293 inducible stable cell lines expressing Cavβ2e-mRFP and Cavβ2e-L9A-mRFP. To obtain the pcDNA5TM/FRT/TO Cavβ2e-mRFP construct, the full-length Cavβ2e cDNA was amplified through PCR using the pcDNA3.1 Cavβ2e-YFP vector, while the mRFP fragment was amplified from the pcDNA3.1 mRFP vector. Subsequently, both PCR fragments were inserted in-frame into the pcDNA5TM/FRT/TO vector using conventional molecular biology techniques. In a similar manner, the pcDNA5TM/FRT/TO Cavβ2e-L9A-mRFP construct was prepared by following the same approach as the pcDNA3.1 Cavβ2e-YFP mutants, but utilizing the pcDNA5TM/FRT/TO Cavβ2e-mRFP vector as the template.

The cDNAs encoding wild-type Cavβ2e-NTv or mutant versions were subcloned into PGEX 6P-1 vector using standard PCR methods to produce GST-Cavβ2e-NTv domain fusion constructs.

### Transient transfections of tsA201 cells

The tsA201 cells were cultured on plastic μ-dishes (Ibidi) in Dulbecco’s modified Eagle’s medium (DMEM) supplemented with 10% fetal bovine serum and L-glutamine (2 mM), and were maintained in a 5% CO_2_-humidified atmosphere. Transient transfections of the cells with the wild-type or mutants Cavβ2e constructs or with the Cavβ2e-NTv-YFP_3_ variants were conducted using Lipofectamine™ 2000 (Invitrogen. Cat. Num. 11668027) following the guidelines provided by the manufacturer. For each construct, a minimum of three transfections were performed to ensure reproducibility and reliability of the results.

### Preparation of Cavβ2e stable cell lines

To generate inducible stable cell lines expressing the Cavβ2e-mRFP or Cavβ2e-L9A-mRFP constructs, Flp-In™ T-REx™-293 cells were seeded at a density of 30%–40% in DMEM supplemented with 10% fetal bovine serum and 2 mM L-glutamine. Cells were co-transfected with 9 µg of the pOG44 plasmid and 1 µg of either pcDNA5™/FRT/TO Cavβ2e-mRFP or pcDNA5™/FRT/TO Cavβ2e-L9A-mRFP constructs using Lipofectamine, following the manufacturer’s protocol. After 24 h, the culture medium was replaced to remove transfection agents, allowing the cells to grow without selection antibiotics. Fresh medium containing Hygromycin B (100 μg/mL) and Blasticidin (10 μg/mL) was added after an additional 24 h. The medium was subsequently changed every 3–4 days until single colonies formed. These colonies were then picked and transferred to multi-well plates for expansion and analysis. Expression of the constructs was induced by adding tetracycline (6 μg/mL) to fresh medium and incubating the cells for 18–24 h. Analysis of expression levels in the different clones was performed by confocal microscopy as described below. The entire process took approximately 21–28 days.

### Confocal microscopy and colocalization analysis

In tsA201 cells cells transiently expressing wild-type Cavβ2e-YFP or its mutants, live-cell imaging was performed using an inverted Leica confocal microscope with a ×63 oil immersion objective. Images were acquired 24–30 h post-transfection. Prior to imaging, cells were stained with the nuclear marker DRAQ5™ (ThermoFisher Scientific) according to the manufacturer’s instructions. YFP fusion proteins were excited using a 514-nm laser, and emission was detected in the range of 520–550 nm. The DRAQ5™ was excited at 633 nm, and emission was monitored in the range of 640–700 nm. Colocalization analysis was carried out using the Mander´s overlap coefficient (MOC) calculated using the JACoP plugin integrated into ImageJ 1.44p software (National Institutes of Health, Bethesda) (Miranda-Laferte et al., 2014; Schneider et al., 2012). At least five different fields of view, comprising a total of 150–250 transfected cells, obtained from a minimum of three independent transfections, were analyzed for each Cavβ2e mutant. The MOC represents the proportion of YFP channel pixels overlapping with the DRAQ5™ detection channel signal. Statistical comparison between samples was performed using a two-tailed Student’s t-test. All values are presented as mean ± standard error (S.E.).

Cells transiently expressing wild-type Cavβ2e-YFP were also incubated for 60 min with 3 µM ionomycin or dimethyl sulfoxide (DMSO) as controls at 37°C, 20–24 h post-transfection. Subsequently, live cell imaging and analysis were performed following previously described methods.

Live cell imaging was performed on the Cavβ2e-mRFP-expressing stable cell line also using an inverted Leica confocal microscope equipped with a ×63 oil immersion objective. Cavβ2e protein expression was induced with 6 μg/mL of tetracycline 18–24 h prior to imaging, and cells were subsequently stained with the nuclear marker Nucspot® (Biotium) following the manufacturer’s instructions. Cells were then treated with 30 µM of the agonist of PLC m-3M3FBS (Sigma Aldrich) for 60 min at room temperature. Confocal fluorescent cell images were captured every 15 min. Excitation of the Cavβ2e-mRFP fusion protein was carried out using a 595-nm laser, and emission was detected within the 600–630 nm range. The Nucspot® nuclear marker was excited at 488 nm, with emission monitored between 495 and 520 nm. Colocalization and statistical analyses were conducted following the same methods as described for the transient transfection experiments.

### Protein purification

Wild-type and mutants GST-Cavβ2e-NTv fusion proteins were expressed in bacteria and purified using affinity and size-exclusion chromatography as described previously (Hidalgo et al., 2006).

### Liposome preparation

Liposomes were prepared as previously described (Miranda-Laferte et al., 2014). Unilamellar phospholipid vesicles were generated by extruding multilamellar vesicles through polycarbonate filters with a pore size of 100 nm using a handheld extruder (LiposoFast, Avestin, Inc.). 1-Palmitoyl-2-oleoyl-sn-glycerophosphocholine (PC, Avanti 850,457), either alone or mixed with 1-palmitoyl-2-oleoyl-sn-glycero-3-phospho-L-serine (PS, Avanti 840,034) in a 1:3 (PS/PC) molar ratio, was dried under a nitrogen stream. Any remaining solvent was eliminated under vacuum. The resulting lipid film was then rehydrated to a final concentration of 1.5 mM in a buffer containing 50 mM HEPES (pH 7.5) and 50 mM NaCl. To ensure proper lipid dispersion, the suspension underwent three to five freeze-thaw cycles using liquid nitrogen and a 37°C water bath. The processed lipid mixture was extruded to generate large unilamellar vesicles (LUVs) with a uniform diameter of approximately 100 nm for protein-liposome cosedimentation assays.

### Protein-liposome cosedimentation assays

Cosedimentation assays were performed as described previously (Miranda-Laferte et al., 2014). In brief, 6.0 µg of GST-Cavβ2e-NTv proteins were incubated with PS/PC or PC alone unilamellar vesicles in a buffer containing 50 mM HEPES and 50 mM NaCl (pH 7.5), in a final volume of 100 μL, for 15 min at room temperature. The samples were then ultracentrifuged at 150,000 × g for 40 min at 4 °C using a TLA 120.1 rotor (Beckman Instruments). The resulting pellets were resuspended in a volume equal to that of the supernatant, and equal volumes from each sample were analyzed by SDS-PAGE (12%). Protein levels in the different fractions were quantified by densitometric analysis of Coomassie Blue-stained gels using ImageJ software (Schneider et al., 2012). The fraction of protein bound to liposomes was determined as the ratio of non-aggregated protein in the pellet to the total non-aggregated protein, as previously described (Miranda-Laferte et al., 2014).

### Quantitative mass spectrometry analyses

For mass spectrometry (MS) analysis, two stable Flp-In™ T-REx™-293 cell lines expressing Cavβ2e-mRFP or Cavβ2e-L9A-mRFP were utilized. Cells were seeded in 10 cm dishes and cultured to approximately 90% confluence. They were then treated with tetracycline (1 μg/mL) or DMSO (negative control) for 24 h. After treatment, cells were collected by scraping into PBS, followed by centrifugation at 3,000 rpm. The resulting cell pellets were flash-frozen in liquid nitrogen and stored at −80°C until further analysis. As an additional negative control, parental Flp-In™ T-REx™-293 cells, which do not express any exogenous genes, were also treated with tetracycline and DMSO under identical conditions. Each of the six experimental conditions was performed in five independent replicates to ensure reliability and reproducibility.

For MS analysis cell pellets were lysed in urea buffer (7M urea, 2M Thiourea, 50 mM Tris, pH 8) in an ultrasonication bath. 10 μg of cell protein (as determined using Bio-Rad Protein Assay Dye Reagent, Bio-Rad Laboratories, United States, Cat# 5000006) were reduced in 10 mM dithiothreitol, alkylated with 15 mM iodoacetamide and digested with trypsin (SERVA Electrophoresis, Heidelberg, Germany) in 1:50 enzyme-to-substrate ratio at 37 °C overnight. The digestion was terminated by the addition of trifluoroacetic acid at a concentration of 0.1%.

200 ng of each cell digest were injected and pre-concentrated using a Vanquish Neo UHPLC system (Thermofisher Scientific, Bremen, Germany) with a trap column (Acclaim PepMap 100, 300 μm × 5 mm, C18, 5 μm, 100 Å). The peptides were then separated on an analytical column (Acclaim PepMap RSLC, 75 μm × 15 cm, nano Viper, C18, 2 μm, 100 Å) at a flow rate of 400 nL/min with the solvent gradient from 1% B to 21% B (B: 84% acetonitrile, 0.1% FA) for 70 min, with a subsequent increase up to 40% for 25 min and washing for 5 min with 95% B. The separated peptides were ionized by electrospray ionization (ESI) and subsequently introduced into an Orbitrap Exploris 480 mass spectrometer (Thermofisher Scientific, Bremen, Germany). The capillary temperature was set to 275 °C, the spray voltage to 1550 V and the RF to 50%. Full MS spectra were acquired in the range from 375 to 1,500 m/z with a resolution of 120,000 at 200 m/z (AGC target 250%, maximum injection time 150 ms). The 25 most intense ions (charge state +2 to +7) were selected for fragmentation with high-energy collision-induced dissociation (HCD) employed to generate the MS/MS fragments. The normalized collision energy (NCE) was set at 31. Intensity threshold was set to 1.0e04, dynamic exclusion to 80 s. The fragments were analyzed in an Orbitrap mass analyzer with a 30,000 resolution (isolation window 0.8 m/z, AGC target 150%, maximum injection time 50 ms).

The suitability of the generated data for quantitative analysis was assessed using the previously published in-house quality control tool, MaCProQC (Rozanova et al., 2023). Based on the quality control one sample (namely Cavβ2e-L9A-mRFP-NI replicate 2) was excluded from the further quantitative analysis. The mass spectrometry proteomics data from the DDA analysis were deposited at the ProteomeXchange Consortium via the PRIDE partner repository (Perez-Riverol et al., 2019), with the data set identifiers PXD058727 and 10.6019/PXD058727.

Obtained raw files were analyzed using MaxQuant (v.2.4.9.0, https://maxquant.org/, accessed on 29 October 2024). Spectra were searched against the human reference proteome (UP000005640) of the UniProtKB database (release 2021_11) using the following parameters: enzyme = trypsin, maximum missed cleavages = 2. Methionine oxidation was set as variable modifications; cysteine carbamidomethylation as fixed. Instrument settings were left as default. PSM identification was performed using a reversed decoy-based false discovery rate of 0.01. The minimum number of peptides and razor peptides for protein identification was 1; the minimum number of unique peptides was 0. Protein identification was performed at a protein false discovery rate of 0.01. The “match between runs” option was disabled. Proteins were quantified with MaxQuant label-free quantification (LFQ)], with unique peptides at the minimum ratio count of 2. The “classic” normalization was applied to the LFQs. The protein quantification results from MaxQuant were analyzed using Perseus 1.6.14.0 (Tyanova et al., 2016). For this the ProteinGroups.txt file from the MaxQuant output was subjected to processing in Perseus, and the resulting LFQs were investigated. The PG “only identified by site” and “Reverse” were excluded from further analysis. Missing LFQ values (where LFQ = 0) were appropriately addressed by substituting them with NA. The LFQs were subsequently transformed using the log2 function, and the quality of the normalization process and inter- and intra-group differences were investigated using boxplots and principal component analysis (PCA). For PCA, only those proteins which had not been assigned a missing LFQ intensity value were included. A differential analysis of protein levels between the corresponding conditions was conducted using a two-sided t-test at a significance level of p = 0.01, with the false discovery rate (FDR) employed for correction of multiple testing and fold change of 2. The t-test results were visualized using R version 4.4.1(R Core Team, 2021) using ‘EnhancedVolcano’ version 1.22.0 Package [https://github.com/kevinblighe/EnhancedVolcano.].

## Results

### PLC activation induces a homogeneous distribution of membrane-associated Cavβ2e between the cytoplasm and nucleus

Activation of the M1 muscarinic GPCR triggers PLC signaling cascade and promotes the release of Cavβ2e from the plasma membrane (Kim et al., 2015b). Here, we bypassed GPCR activation and analyzed the cellular distribution of Cavβ2e following pharmacological PLC stimulation. We established a stable Flp-In™ T-REx™-293 cell line expressing Cavβ2e fused to mRFP. The cells were loaded with NucSpot® nuclear marker, exposed for 60 min to 30 µM of the PLC agonist m-3M3FBS, and imaged using laser scanning confocal microscopy. The levels of nuclear localization of the protein were evaluated by calculating the ratio of nuclear versus total protein using the Mander´s overlap coefficient.

**FIGURE 1:**
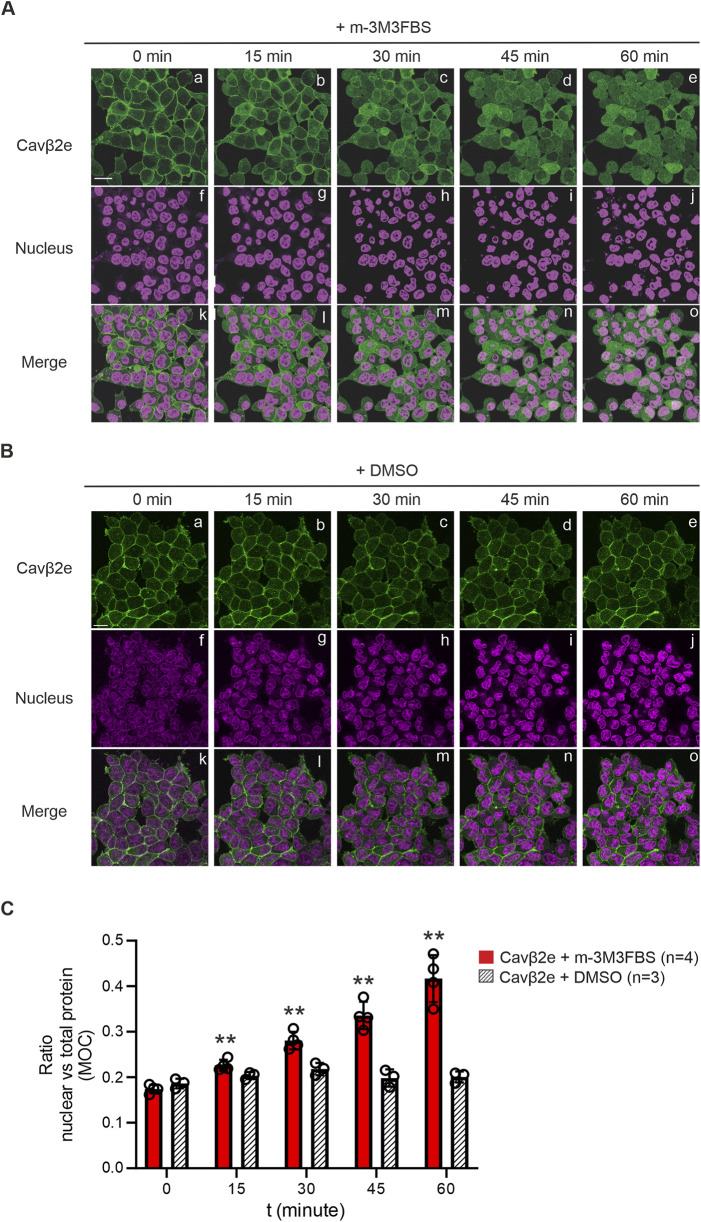
Membrane-targeted Cavβ2e redistributes evenly between the cytosol and nucleus upon PLC activation. **(A)** Representative confocal fluorescence images of a stable Flp-In™ T-REx™-293 cell line expressing Cavβ2e fused to mRFP and stained with the Nucspot® nuclear marker. Cells were treated with the PLC agonist m-3M3FBS for the indicated time points. The fluorescence signal for Cavβ2e (panels a–e), for the nuclear marker (panels f–j), and merged images (panels k–o) are shown. The scale bar (15 µm) applies to all images. **(B)** Representative confocal fluorescence images of cells as in A but treated with DMSO as negative control. The fluorescence signal for Cavβ2e (panels a–e), for the nuclear marker (panels f–j), and merged images (panels k–o) are shown. The scale bar (15 µm) applies to all images **(C)** Plot of the colocalization analysis from **(A, B)**, between Cavβ2e and the Nucspot® nuclear marker based on the Mander´s overlap coefficient (MOC). Values are presented as mean ± S.E. **p < 0.01 vs t = 0 min (two-tailed Student’s t-test).

Untreated cells showed the characteristic membrane localization of Cavβ2e (Colecraft et al., 2002; Kim et al., 2015a; Miranda-Laferte et al., 2014), but we also observed a small fraction of the protein located in the nucleus (Figure 1A). With increasing incubation times with m-3M3FBS, Cavβ2e was steadily released from the plasma membrane and showed a significant time-dependent increase in cytosolic and nuclear localization (Figures 1A, C), an effect that was not observed in vehicle-treated cells (Figures 1B, C). After 60 min of PLC activation, virtually no signal from Cavβ2e was visible at the plasma membrane, and the protein was homogeneously distributed between the cytosol and the nucleus (Figures 1A, C). This indicates that the increase in cytosolic Cavβ2e following its PLC-mediated release from the plasma membrane promotes an increase in nuclear Cavβ2e levels.

### Intracellular calcium mediates Cavβ2e dissociation from the plasma membrane

As previously mentioned, PLC activation leads to PIP2 cleavage at the plasma membrane, increasing intracellular Ca^2+^ levels. This process can directly detach Cavβ2e molecules bound to PIP2. Additionally, the rise in intracellular calcium may weaken electrostatic interactions between the Cavβ2e-NTv domain and phospholipids in the membrane bilayer, promoting the release of the protein from the plasma membrane and triggering its redistribution within the cell.

**FIGURE 2:**
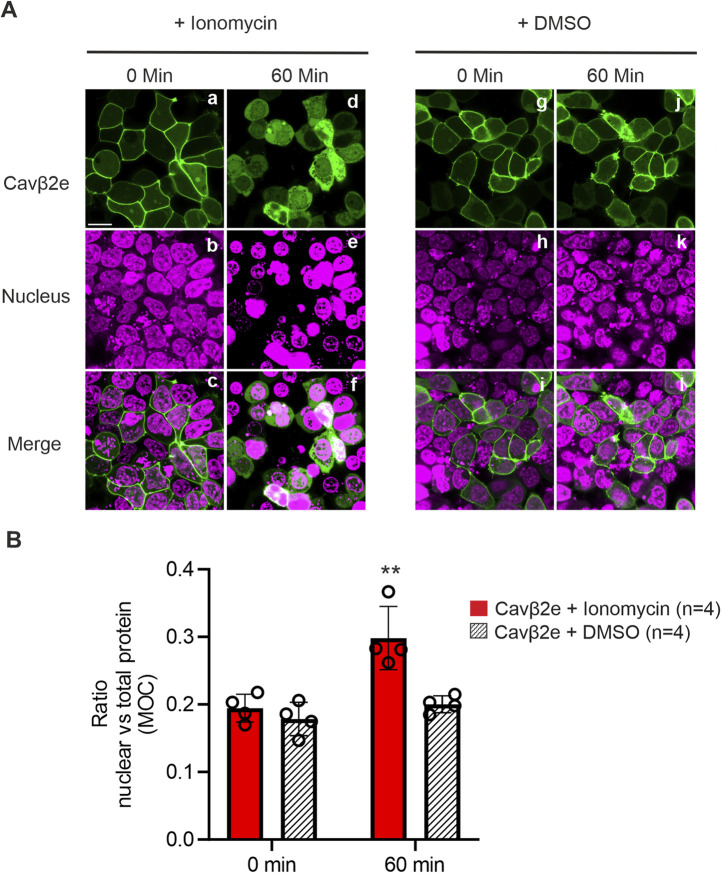
Ca^2+^ mediates PLC-induced cellular redistribution of membrane-targeted Cavβ2e. **(A)** Confocal fluorescence images of tsA201 cells expressing Cavβ2e fused to YFP and untreated or treated with either ionomycin or DMSO (negative control) for 60 min. The fluorescence signal for Cavβ2e (panel a, d, g and j), DRAQ5™ nuclear marker (panel b, e, h and k), and merged images (panels c, f, i and l) are shown. The scale bar (15 µm) is applicable to all images. **(B)** Plot illustrating the colocalization analysis from panel A between Cavβ2e and the DRAQ5™ marker based on the Mander´s overlap coefficient (MOC). All values are presented as mean ± S.E. **p < 0.01 vs untreated wild-type Cavβ2e (two-tailed Student’s t-test).

To elucidate the mechanism underlying Cavβ2e dissociation from the plasma membrane upon PLC activation, we used the calcium ionophore ionomycin to assess the role of intracellular calcium in this process. Cells transiently expressing wild-type Cavβ2e-YFP were treated with ionomycin or vehicle and analyzed by laser scanning confocal microscopy. After 60 min of treatment, Cavβ2e was fully released from the plasma membrane and exhibited a homogeneous distribution between the cytosol and nucleus, an effect not observed in vehicle-treated cells (Figures 2A, B). This cellular distribution resembles that observed following PLC activation. These results suggest that the intracellular Ca^2+^ increase induced by PLC activation facilitates Cavβ2e redistribution from the plasma membrane to the cytosol and nucleus.

### The N-terminus of Cavβ2e contains a functional nuclear localization signal

**FIGURE 3:**
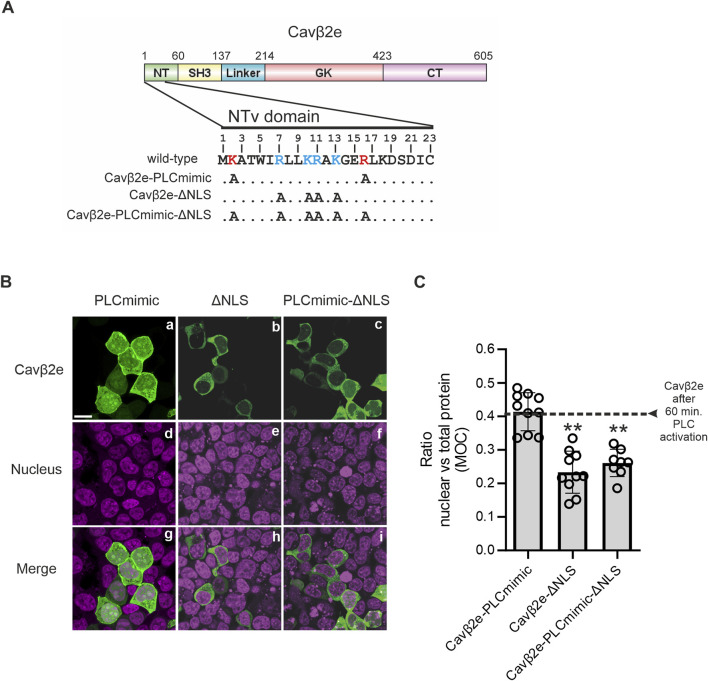
Cavβ2e contains a NLS within the NTv domain. **(A)** Schematic representation of the domain structure of Cavβ2e. NT (N-terminus), SH3 (Src-Homology 3 domain), GK (Guanylate-kinase-like domain), and CT (C-terminus) are indicated. The 23-amino-acid-long sequence of the NTv domain of Cavβ2e that distinguishes Cavβ2e from all other Cavβ2 splice variants is shown. The residues Lys2 and Arg16, which, when mutated to alanine, mimic the effect of PLC activation, are highlighted in bold red. Positively charged amino acids identified as part of the nuclear localization signal are marked in bold blue. Alanine mutations performed to generate the constructs Cavβ2e-PLCmimic, Cavβ2e-ΔNLS, and Cavβ2e-PLCmimic-ΔNLS are also indicated. **(B)** Representative confocal fluorescence images of tsA201 cells expressing the indicated Cavβ2e mutants fused to YFP and stained with the DRAQ5™ nuclear marker. The fluorescence signal for Cavβ2e (panels a–c), the nuclear marker (panels d–f), and merged images (panels g–i) are shown. The scale bar (15 µm) applies to all images. **(C)** Plot of the colocalization analysis from panel B, showing the overlap between the Cavβ2e mutants and the DRAQ™ nuclear marker based on Mander’s overlap coefficient (MOC). The black dashed line represents the average value obtained for wild-type Cavβ2e after a 60-min treatment with the PLC agonist m-3M3FBS, as shown in Figure 1C. Values are presented as mean ± S.E. **p < 0.01 vs. Cavβ2e-PLCmimic (two-tailed Student’s t-test).

To study the molecular determinants of Cavβ2e nuclear translocation, we analyzed the cellular distribution of mutants with impaired plasma membrane association to mimic the release of the protein from the cell surface upon PLC activation. We have previously demonstrated that alanine mutations in positively charged amino acids within the NTv region of Cavβ2e lead to its dissociation from the plasma membrane (Miranda-Laferte et al., 2014). Here, we found that one of these mutant proteins, Cavβ2e K2A, R16A (Cavβ2e-PLCmimic; Figure 3A and Table 1), exhibited equal distribution between the cytosol and the nucleus, comparable to that observed for wild-type Cavβ2e after 60 min of PLC activation with m-3M3FBS (Figures 3B, C). In contrast, another non-membrane-associated alanine mutant, Cavβ2e R7A, K10A, R11A, K13A, exhibited significantly reduced nuclear localization compared to the wild-type protein following PLC activation, as well as to Cavβ2e-PLCmimic (Figures 3B, C). This suggests that residues Arg7, Lys10, Arg11, and Lys13 are involved in Cavβ2e nuclear targeting. Furthermore, using cNLS Mapper (Kosugi et al., 2009) to predict nuclear localization signals (NLSs) within the N-terminal segment of Cavβ2e, we obtained a relatively high score for the segment spanning residues 7 to 15 (7-RLLKRAKGE-15). Hereafter, we refer to the Cavβ2e R7A, K10A, R11A, K13A mutant as Cavβ2e-ΔNLS (Figure 3A; Table 1). Therefore, mutations of those NLS critical residues into the background of Cavβ2e-PLCmimic (Cavβ2e-PLCmimic-ΔNLS, Figure 3A; Table 1) are expected to exhibit reduced nuclear distribution compared to Cavβ2e-PLCmimic. Indeed, Cavβ2e-PLCmimic-ΔNLS showed decreased nuclear versus total protein ratio, confirming the involvement of residues Arg7, Lys10, Arg11, and Lys13 as part of a NLS at the NTv of Cavβ2e (Figures 3B, C).

**TABLE 1:** Summary of the different Cavβ2e derivate constructs used in this stud**y**. The Mander’s overlap coefficient (MOC) represents the ratio of nuclear to total protein for each construct and is expressed as means ± S.E.

Construct name	Mutations in Cavβ2e	MOC
Cavβ2e	n.a	0,19 ± 0,02
Cavβ2e-PLCmimic	K2A, R16A	0,41 ± 0,02
Cavβ2e-ΔNLS	R7A, K10A, R11A, K13A	0,23 ± 0,02
Cavβ2e-PLCmimic-ΔNLS	K2A, R7A, K10A, R11A, K13A, R16A	0,26 ± 0,01
Cavβ2e-PLCmimic-L8A	K2A, L8A, R16A	0,57 ± 0,02
Cavβ2e-PLCmimic-L9A	K2A, L9A, R16A	0,49 ± 0,02
Cavβ2e-ΔNLS-L8A	R7A, L8A, K10A, R11A, K13A	0,32 ± 0,04
Cavβ2e-ΔNLS-L9A	R7A, L9A, K10A, R11A, K13A	0,27 ± 0,02
Cavβ2e-L8A	L8A	0,45 ± 0,02
Cavβ2e-L9A	L9A	0,52 ± 0,03
Cavβ2e-W5A	W5A	0,31 ± 0,02
YFP_3_	n.a	0,23 ± 0,01
NTv-YFP_3_	n.a	0,21 ± 0,02
NTv-PLCmimic-L9A-YFP_3_	K2A, L9A, R16A	0,48 ± 0,03
NTv-ΔNLS-L9A-YFP_3_	R7A, L9A, K10A, R11A, K13A	0,25 ± 0,01

### A dileucine motif in the N-terminus of Cavβ2e acts as a nuclear export signal

Cytosol-nuclear shuttling proteins require a nuclear export signal (NES) to traffic from the nucleus to the cytosol. Canonical NESs typically consist of a stretch of hydrophobic residues, often enriched with leucine residues and interspersed with other amino acids. The NTv domain of Cavβ2e contains several leucine and isoleucine residues that could potentially serve as an NES. Using NetNES (La Cour et al., 2004) to predict NESs within the N-terminal segment of Cavβ2e yielded a relatively high score for the segment spanning residues 8 to 17 (8-LLKRAKGERL-17) containing the dileucine, Leu8-Leu9, motif. Therefore, we decided to experimentally evaluate whether these residues are involved in Cavβ2e nucleus-cytosol trafficking.

**FIGURE 4:**
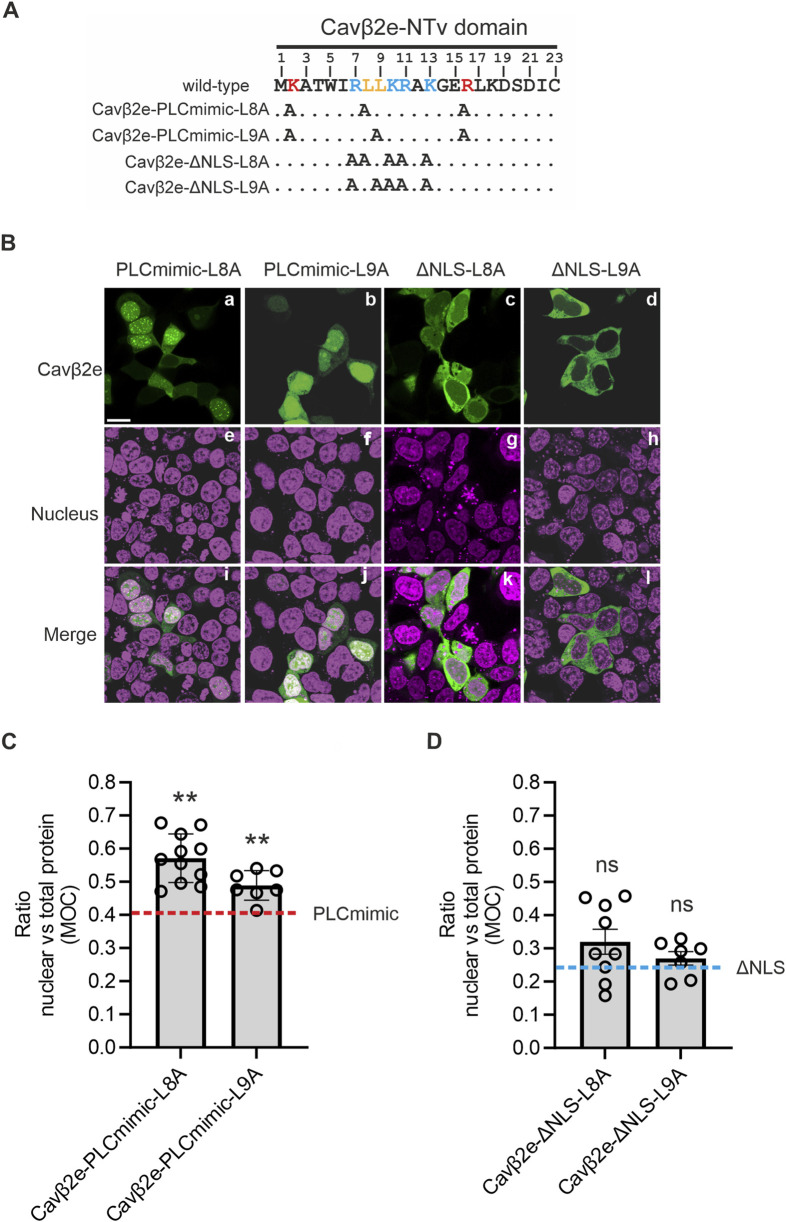
A NES at Cavβ2e-NTv domain is necessary to export the protein from the nucleus. **(A)** The 23-amino-acid-long sequence of the NTv region Cavβ2e, corresponding to the variable NTv domain that distinguishes Cavβ2e from all other Cavβ2 splice variants, is shown. The dileucine motif, identified as a nuclear export signal, is highlighted in bold orange. The residues Lys2 and Arg16, which, when mutated to alanine, mimic the effect of PLC activation, are highlighted in bold red. Positively charged amino acids identified as part of the nuclear localization signal are marked in bold blue. Alanine mutations in the constructs Cavβ2e-PLCmimic-L8A, Cavβ2e-PLCmimic-L9A, Cavβ2e-ΔNLS-L8A, and Cavβ2e-ΔNLS-L9A are indicated. **(B)** Representative confocal fluorescence images of tsA201 cells expressing the indicated Cavβ2e mutants fused to YFP and stained with the DRAQ5™ nuclear marker. The fluorescence images for Cavβ2e mutant proteins (panels a–d), the DRAQ5™ nuclear marker (panels e–h), and merged images (panels i–l) are shown. The scale bar (15 µm) applies to all images. **(C)** Plot of the colocalization analysis from panel B between the mutants Cavβ2e-PLCmimic-L8A, Cavβ2e-PLCmimic-L9A, and the DRAQ5™ nuclear marker, based on Mander’s overlap coefficient (MOC). The red dashed line represents the average value obtained for the Cavβ2e-PLCmimic mutant, as shown in Figure 3C. **(D)** Plot displaying the colocalization analysis results from panel B between the mutants Cavβ2e-ΔNLS-L8A and Cavβ2e-ΔNLS-L9A with the DRAQ5™ nuclear marker, based on Mander’s overlap coefficient (MOC). The blue dashed line represents the average value obtained for the Cavβ2e-ΔNLS mutant, as shown in Figure 3C. All values are presented as mean ± S.E. ^**^p < 0.01 vs. Cavβ2e-PLCmimic (two-tailed Student’s t-test); ns, non-significant vs. Cavβ2e-ΔNLS.

We inserted as single mutations L8A and L9A into the background of the Cavβ2e-PLCmimic construct. The resulting generated constructs (Cavβ2e-PLCmimic-L8A; and Cavβ2e-PLCmimic-L9A; Figure 4A; Table 1) exhibited a significant higher nuclear accumulation compared to Cavβ2e-PLCmimic (Figures 4B, C). These results suggest that Leu8 and Leu9 are critical components of an NES in Cavβ2e.

To rule out the possibility that the increased nuclear localization of Cavβ2e-PLCmimic-L8A and Cavβ2e-PLCmimic-L9A mutants resulted from enhanced nuclear import, we introduced the same L8A and L9A mutations into the Cavβ2e-ΔNLS construct, which lacks the NLS critical residues. The resulting mutants (Cavβ2e-ΔNLS-L8A and Cavβ2e-ΔNLS-L9A; Figure 4A; Table 1) are excluded from the nucleus to the same extent as Cavβ2e-ΔNLS (Figures 4B, D). This finding demonstrated that the increased nuclear accumulation of the L8A and L9A mutants in the Cavβ2e-PLCmimic background was due to impaired nuclear export rather than enhanced nuclear import.

**FIGURE 5:**
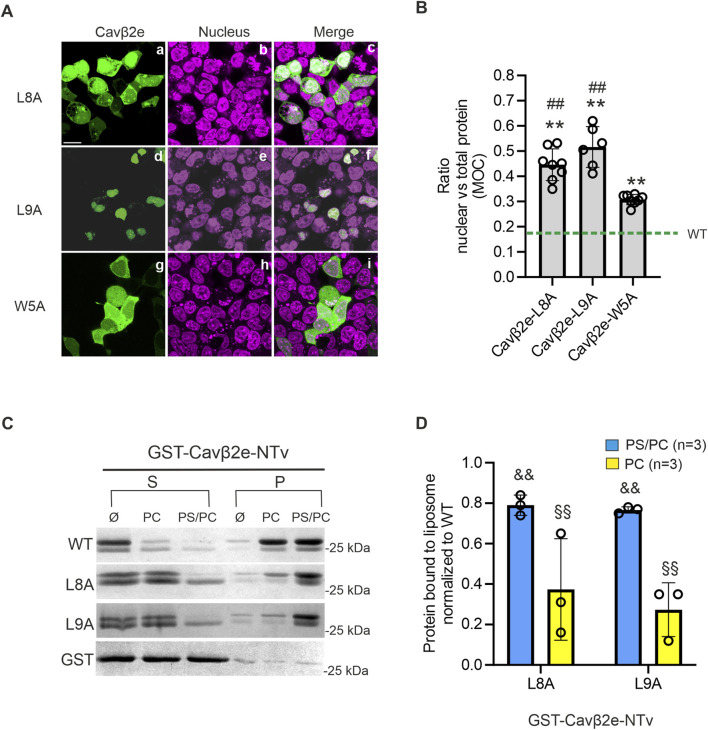
The dileucine motif at the NTv domain of Cavβ2e is involved in the nuclear export and the plasma membrane anchoring of the protein. **(A)** Representative confocal fluorescence images of tsA201 cells expressing the indicated Cavβ2e mutants fused to YFP and stained with the DRAQ5™ nuclear marker. The fluorescence images for Cavβ2e mutant proteins (panels a, d and g), DRAQ5™ nuclear marker (panels b, e, and h), and merged images (panels c, f, and i) are shown. The scale bar (15 µm) applies to all images. **(B)** Plot displaying the colocalization analysis results from panel A between the Cavβ2e mutants and the DRAQ5™ nuclear marker, based on Mander’s overlap coefficient (MOC). The green dashed line represents the average value obtained for the untreated wild-type Cavβ2e (WT), as shown in Figure 2B. **(C)** Representative SDS-PAGE gels from protein-liposome cosedimentation assays using wild-type and mutants Cavβ2e-NTv fused to GST (GST-Cavβ2e-NTv). Supernatant (S) and pellet (P) fractions after centrifugation of the corresponding GST-Cavβ2e-NTv protein in assay buffer only (Ø) or preincubated with either neutral (PC) or negatively charged (PS/PC) liposomes. Controls assays using the GST moiety alone were performed in parallel and are also shown. **(D)** Plot of the fraction of GST-Cavβ2e-NTv mutant proteins bound to either PS/PC or PC liposomes. The data are normalized to the WT values. All values are presented as mean ± S.E. **p < 0.01 vs wild-type Cavβ2e; ^##^p < 0.01 vs. Cavβ2e W5A; ^&&^ p < 0.01 vs. wild-type GST-Cavβ2e-NTv in PS/PC and ^§§^p < 0.01 vs. wild-type GST-Cavβ2e-NTv in PC (two-tailed Student’s t-test).

When we introduced the L8A and L9A mutations into wild-type Cavβ2e, we observed a predominantly lack of membrane localization in cells expressing these mutants. In many cells expressing the L8A mutant, the protein accumulated in the nucleus, whereas in cells expressing the L9A mutant, it was almost entirely sequestered in this compartment (Figures 5A, B; Table 1).

Since the Cavβ2e L8A and L9A mutants do not appear to be targeted to the plasma membrane, we assessed their affinity for lipid bilayers using protein-liposome cosedimentation assays. The WT, L8A, and L9A NTv domains of Cavβ2e were fused to GST (GST-Cavβ2e-NTv), expressed in bacteria, purified, and incubated with liposomes containing either negatively charged (PS/PC) or neutral (PC) phospholipids. After centrifugation, the supernatant and pellet fractions, containing lipid-free and lipid-associated protein, respectively, were analyzed using SDS-PAGE. To rule out the possibility that the protein observed in the pellet resulted from an interaction between GST and the lipids, we conducted parallel experiments in which GST-Cavβ2e-NTv was replaced with GST alone. In all conditions, GST was virtually absent from the pellet after centrifugation (Figure 5C). We observed a significant reduction of 25% and 70% in affinity for negatively charged and neutral phospholipids, respectively, in both mutants compared to WT (Figures 5C, D). These results confirm that the Leu8 and Leu9 residues are not only part of the NES but also play a role in Cavβ2e membrane targeting.

It has been previously demonstrated that another hydrophobic residue (Trp5) also plays a role in Cavβ2e membrane targeting (Kim et al., 2015a; Miranda-Laferte et al., 2014). Since our results show that the Leu8 and Leu9 residues have a dual role in nuclear trafficking and membrane localization, we also analyze the nuclear distribution of the Cavβ2e W5A mutant. Cells expressing Cavβ2e W5A exhibited a significantly lower nuclear distribution compared to the L8A and L9A mutants (Figures 5A, B; Table 1), suggesting that Trp5 is not part of an NES.

### The N-terminus of Cavβ2e suffices for nuclear targeting

The NTv domain of Cavβ2e has been established as a sufficient determinant for protein attachment to the plasma membrane (Miranda-Laferte et al., 2014). We next investigated whether this segment suffices for nuclear transport, independent of other Cavβ2e regions. To address this, we fused both wild-type and mutated Cavβ2e-NTv domain with YFP. Given that a the fusion of the NTv region to a single YFP molecule results in a construct with a molecular mass of approximately 27 kDa, and proteins with a molecular weight below 60 kDa are known to diffuse passively through nuclear pores without specific signals (Timney et al., 2016), we developed a strategy to exceed this threshold. To increase the molecular mass beyond 60 kDa, we fused the Cavβ2e-NTv domain to a three-YFP tandem (YFP_3_), resulting in the Cavβ2e-NTv-YFP_3_ constructs.

**FIGURE 6:**
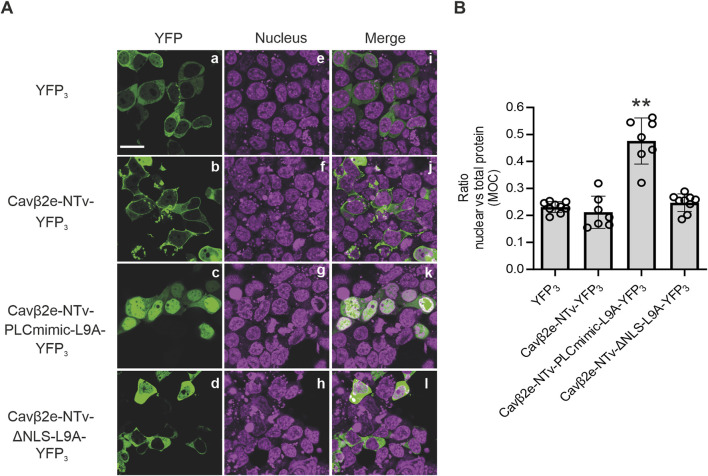
Cavβ2e N-terminus suffices to control the plasma membrane-nuclear shuttling of the protein. **(A)** Representative confocal fluorescence images of tsA201 cells expressing either wild-type or the indicated mutant Cavβ2e-NTv domain fused to a three-tandem YFP molecules (YFP_3_). Cells expressing YFP_3_ alone were used as controls. The fluorescence signal for YFP_3_-fused proteins (panels a–d), nucleus (panels e–h), and merged images (panels i–l) are shown. The scale bar (15 µm) applies to all images. **(B)** Plot displaying the colocalization analysis from panel B between YFP_3_-fused proteins and the DRAQ5™ nuclear marker based on Mander’s overlap coefficient (MOC). Values are presented as mean ± S.E. **p < 0.01 vs. YFP_3_ (two-tailed Student’s t-test).

As expected, the control YFP_3_ tandem expressed in cells exhibited a cytosolic localization, while the wild-type Cavβ2e-NTv-YFP_3_ construct displayed prominent protein distribution along the plasma membrane (Figures 6A, B; Table 1). The Cavβ2e-NTv-PLCmimic-L9A-YFP_3_ and Cavβ2e-NTv-ΔNLS-L9A-YFP_3_ mutants (Table 1) display the predominantly nuclear and cytosolic subcellular distribution, respectively, as their full-length Cavβ2e-PLCmimic and Cavβ2e-ΔNLS protein counterparts (Figures 6A, B). These findings demonstrate that both the NLS and the NES present in the NTv region of Cavβ2e suffices for the nuclear trafficking of the protein.

### Nuclear Cavβ2e regulates gene expression

Several studies have demonstrated that different Cavβ isoforms and splice variants regulate the expression of various genes. Given that Cavβ2e translocates from the plasma membrane to the nucleus following PLC activation, we aimed to determine whether nuclear Cavβ2e could also regulate gene expression in Flp-In™ T-REx™-293 cells. To identify proteins whose expression might be regulated by nuclear Cavβ2e, we compared the proteomes of Flp-In™ T-REx™-293 cells expressing wild-type Cavβ2e and those expressing Cavβ2e-L9A, a mutant highly localized in the nucleus (Figures 5A, B), using quantitative MS analysis. Control samples included Flp-In™ T-REx™-293 cells containing the wild-type Cavβ2e and the Cavβ2e-L9A mutant gene inserts, but not expressing either protein, as they were not induced. These controls were used to account for background proteomic variations.

**FIGURE 7:**
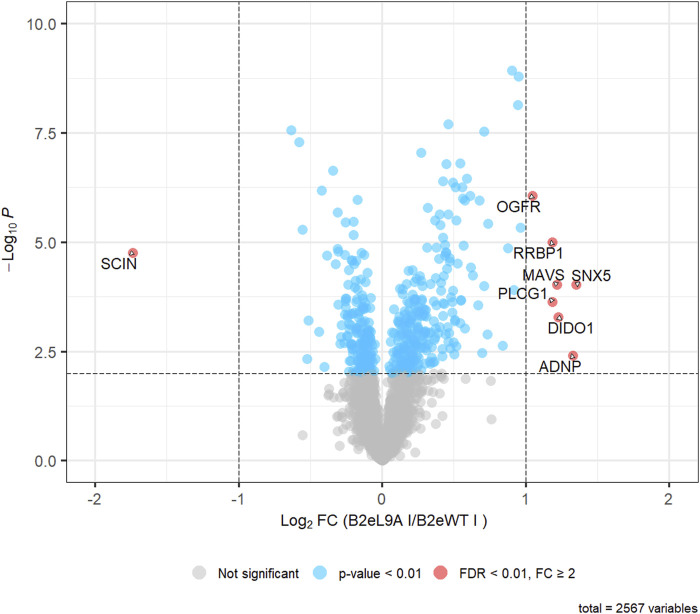
Quantitative MS-analysis. Volcano plot representing the results of a two-tailed Student’s t-test comparing 2,567 protein LFQ intensities between cells expressing Cavβ2e L9A (B2eL9A) and wild-type Cavβ2e (B2eWT). Among these, 459 proteins were regulated with p < 0.01 (shown in blue), and 8 proteins were significantly regulated (FDR-corrected p < 0.01, fold change (FC) > 2) (shown in red).

In total, 3,905 proteins with at least two unique peptides were identified, of which a total of 2,567 protein groups could be quantified. Statistical analysis revealed 659 proteins that were either upregulated or downregulated in cells expressing Cavβ2e L9A compared to cells expressing wild-type Cavβ2e, using a significance threshold of p < 0.01 (Figure 7; Supplementary Table S1). However, applying a stringent threshold of false discovery corrected p-values (FDR) < 0.01 and fold changes (FC) > 2, representing the standard criterion in quantitative MS analysis, the number of significantly different proteins was substantially reduced to eight. These included the following upregulated proteins: opioid growth factor receptor (OGFR), ribosome-binding protein 1 (RRBP1), mitochondrial antiviral-signaling protein (MAVS), sorting nexin-5 (SNX5), 1-phosphatidylinositol 4,5-bisphosphate phosphodiesterase gamma-1 (PLCG1), death-inducer obliterator 1 (DIDO1), and activity-dependent neuroprotector homeobox protein (ADNP). Adseverin (SCIN) was identified as a downregulated protein in cells expressing Cavβ2e-L9A compared to those expressing wild-type Cavβ2e (Figure 7; Supplementary Table S1). Four of these proteins including OGFR, RRBP1, PLCG1, and ADNP were also significantly regulated in the comparison of the control samples and were therefore not distinct to Cavβ2e-expressing cells (Supplementary Table S1).

Despite the low number of proteins found to be upregulated or downregulated by nuclear Cavβ2e in our heterologous expression system, these results suggest that nuclear Cavβ2e may potentially modulate the expression of specific proteins in a system where it is endogenously expressed. This would be consistent with the genetic regulatory roles observed for other Cavβ isoforms in native contexts.

## Discussion

We and others have previously demonstrated that diverse splice variants of Cavβ1, Cavβ2, Cavβ3, and Cavβ4 are targeted to the nucleus in neurons, cardiomyocytes, skeletal muscle, and other cell types. There, they exert their effects on gene regulation, influencing various physiological and pathophysiological processes such as cardiac hypertrophy, neuronal electrical activity, cell proliferation, impaired muscle progenitor cell expansion, aging, epilepsy, and ataxia (Etemad et al., 2014; Pickel et al., 2021; Rima et al., 2017; Tadmouri et al., 2012; Taylor et al., 2014; Vergnol et al., 2022; Zhang et al., 2010).

Here, we study the nuclear localization of the membrane-associated Cavβ2e variant. We show that upon activation of PLC, Cavβ2e becomes released from the plasma membrane and evenly distributed between the cytoplasm and the nucleus.

Cavβ2e nuclear trafficking is governed by the presence of NLSs and NESs within the NTv domain of Cavβ2e. While the presence of NLSs and NESs has been demonstrated for Cavβ3 and Cavβ4 isoforms (Corzo-López et al., 2023; Etemad et al., 2014), to the best of our knowledge, this is the first report of a mechanism responsible for the nuclear translocation of a Cavβ2 splice variant and the occurrence of such a trafficking event in a membrane-associated Cavβ subunit.

Cavβ2e NLS consists of a stretch of at least four positively charged residues, including Arg7, Lys10, Arg11, and Lys13, classifying it as a monopartite classical nuclear localization signal (Lu et al., 2021). These residues also play a crucial role in Cavβ2e membrane targeting, as previously demonstrated (Miranda-Laferte et al., 2014). Therefore, many of the positively charged residues in the NTv domain of Cavβ2e have a dual role in both nuclear trafficking and membrane localization.

The NES in Cavβ2e consists of a dileucine motif (Leu8, Leu9). Our results from single mutations of these residues show that Leu9 plays a more significant role in nuclear trafficking than Leu8, as a single mutation in Leu9 is sufficient for complete sequestration of the protein in the nucleus. Interestingly, mutations in these residues also promote the release of the protein from the plasma membrane. This was confirmed by protein-liposome cosedimentation assays, where the L8A and L9A mutants exhibited a slight decrease in affinity for negatively charged phospholipids, but their association with neutral liposomes was significantly impaired. These findings demonstrate that Leu8 and Leu9 directly contribute to Cavβ2e membrane binding through hydrophobic interactions, possibly by stabilizing the membrane-bound conformation of the protein through penetration into the lipidic core, as previously shown for Trp5 (Kim et al., 2015a; Miranda-Laferte et al., 2014). Our results also indicate that, similar to residues involved in the NLS, the dileucine motif has a dual effect on both nuclear trafficking and membrane targeting.

Numerous studies have documented the translocation of membrane-associated proteins to the nucleus in response to various cellular conditions (Zheng and Jiang, 2022). For example, scaffolding proteins such as ZO-2, casein kinase 2-interacting protein-1 (CKIP-1), Src-family kinases, and NFAT transcription factors have been shown to translocate from the plasma membrane to the nucleus, where they regulate gene expression (Bagnato et al., 2020; Eisenhaber et al., 2011; Li et al., 2014; Liu et al., 2018; Traweger et al., 2003) These proteins may function directly as transcription factors or indirectly influence gene expression by activating other regulatory proteins, thereby modulating a wide array of cellular processes under both physiological and pathophysiological conditions. In this context, Cavβ2e emerges as a candidate signal transductor translocating to the nucleus and modulating gene expression in response to GPCR activation and subsequent PLC signaling.

In the classical signaling pathway, PLC activation by GPCR ligands and hormones leads to the hydrolysis of PIP2 at the plasma membrane, resulting in the production of diacylglycerol and IP3. Subsequently, IP3 is released into the cytosol and binds to IP3 receptors on the endoplasmic reticulum, triggering the release of Ca^2+^ from this intracellular organelle (Lyon et al., 2013; Nakamura and Fukami, 2017). Therefore, the release of Cavβ2e from the plasma membrane following PLC activation could be due to either the direct effect of PLC on PIP2 cleavage or the transient IP3-mediated increase in intracellular Ca^2+^, which dissociates membrane-associated proteins by weakening electrostatic protein-phospholipid interactions (Clarke, 2023). Cavβ2e can bind to liposomes containing PIP2 and consistently, the direct cleavage of PIP2 molecules by PLC has been shown to contribute to the release of Cavβ2e from the plasma membrane (Kim et al., 2015b). However, due to the relatively low concentration of PIP2 compared to other phospholipids at the plasma membrane (Mulgrew-Nesbitt et al., 2006; Niggli, 2001), most membrane-targeted Cavβ2e molecules are likely bound to other phospholipids. Using ionomycin as a calcium ionophore, we have now demonstrated that an increase in intracellular calcium also plays an important role in Cavβ2e plasma membrane release and that the two aforementioned mechanisms likely have a synergistic effect.

The interaction between Cavβ and VGCCs occurs through a hydrophobic pocket within the Cavβ-GK domain and the α-interaction domain of Cavα1, and does not involve the NTv domain of Cavβ2e (Chen et al., 2004; Opatowsky et al., 2004; van Petegem et al., 2004). Consequently, PLC activation is not expected to dissociate Cavβ2e molecules attached to the Cavα1 subunit from the plasma membrane. Hence, in native systems, where Cavβ2e and Cavα1 subunits can coexist, Cavβ2e nuclear translocation could arise from a pool of Cavβ2e molecules not associated with VGCCs. The existence of this pool of Cavβ2e molecules not interacting with VGCCs is likely to occur in different cell types, since, as previously mentioned, some Cavβ isoforms have been localized in the nucleus of diverse cell types, and Cavβ also has some VGCC-independent functions (Belkacemi et al., 2018; Cruz-Garcia et al., 2021; Etemad et al., 2014; Gonzalez-Gutierrez et al., 2007; Hibino et al., 2003; Miranda-Laferte et al., 2011; Pickel et al., 2021; Subramanyam et al., 2009; Taylor et al., 2014; Vergnol et al., 2022).

**FIGURE 8:**
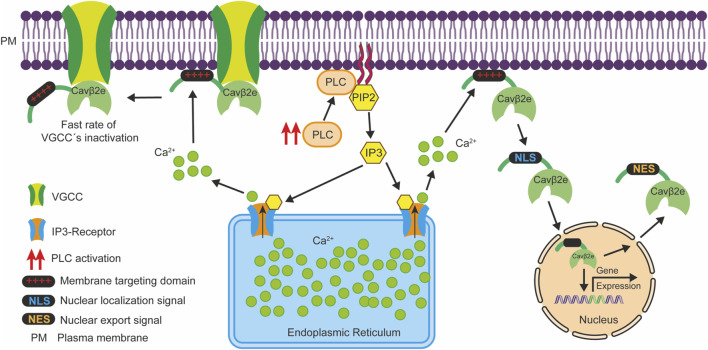
Schematic representation illustrating how PLC signaling potentially exerts a dual physiological effect via Cavβ2e. PLC activation (red arrows) leads to the hydrolysis of PIP2 molecules at the plasma membrane, generating IP3, which triggers Ca^2+^ release from the endoplasmic reticulum through IP3 receptors. This Ca^2+^ release diminishes the electrostatic interactions between the Cavβ2e membrane targeting domain at the NTv region (depicted as red “+++”) and lipids, resulting in two distinct outcomes. First, Cavβ2e molecules that are membrane-associated but not interacting with VGCC are released from the plasma membrane. They then translocate to the nucleus via an NLS at the NTv domain of the protein, and participate in the regulation of gene expression. Subsequently, they can exit the nucleus through an NES, also located within the NTv domain. Second, Cavβ2e molecules that interact with both the plasma membrane and VGCCs contribute to a slower inactivation rate of these channels. In this context, PLC-mediated release of the Cavβ2e-NTv domain from the plasma membrane leads to a faster inactivation rate of the VGCCs as reported by Kim et al. (2015b).

It has been demonstrated that the interaction of the Cavβ2e-NTv domain with the plasma membrane plays an important role in regulating VGCC inactivation (Colecraft et al., 2002; Link et al., 2009; Miranda-Laferte et al., 2014). Accordingly, it has been shown that PLC activation via a muscarinic type 1 receptor agonist regulates the rate of VGCC inactivation by inducing the dissociation of the Cavβ2e-NTv domain from the plasma membrane in VGCC-associated Cavβ2e molecules (Kim et al., 2015b). Therefore, PLC signaling could potentially exert a dual physiological effect through Cavβ2e: one via the regulation of VGCC inactivation through Cavβ2e subunits associated with VGCCs, and another through the regulation of gene expression by promoting the nuclear translocation of Cavβ2e molecules not associated with VGCCs (Figure 8).

Our quantitative mass spectrometry analysis identified a limited number of proteins regulated by Cavβ2e, particularly when using the standard high-stringency threshold typically applied in such analyses. The low number of significantly regulated proteins may be attributed to the heterologous expression system employed (Flp-In™ T-REx™-293 cells), which may lack key factors necessary to assure proper Cavβ2e-mediated gene regulation. Although we cannot directly extrapolate the genetic regulatory function of Cavβ2e to a native system, we believe that Cavβ2e-mediated gene regulation is more extensive in native cells containing the full Cavβ signaling toolkit, as described for other Cavβ isoforms (Etemad et al., 2014; Rima et al., 2017; Tadmouri et al., 2012; Taylor et al., 2014).

Our findings, together with previous studies, highlight the NTv domain of Cavβ2e as a key regulatory hotspot, coordinating multiple functions such as channel activity regulation, plasma membrane targeting, and nuclear trafficking, all of which can be modulated by PLC signaling. Moreover, the overlap of residues involved in these functions further supports the concept that the Cavβ2e-NTv domain acts as a multimodal subcellular targeting signal, integrating these various roles within a single, dynamic structure.

## Limitations of the study

While our study provides evidence for the nuclear localization of Cavβ2e and its potential role in gene regulation, its physiological significance remains to be fully elucidated. Our experiments were conducted in a heterologous expression system, and the extent to which Cavβ2e nuclear translocation occurs in native cells is still unclear. Moreover, the overexpression of Cavβ2e in a heterologous system might not capture the full complexity of Cavβ2e regulation and its downstream effects in more physiologically relevant contexts. Previous studies have reported Cavβ2e expression in various brain regions such as cerebellum, hippocampus, cortex and hypothalamus (Kim et al., 2015b), suggesting a possible physiological function in these brain regions. Therefore, investigating Cavβ2e nuclear translocation and gene regulation in neurons or other brain cells could provide further insights into its relevance in cellular signaling.

Additionally, our study does not directly address the potential pathological implications of Cavβ2e nuclear translocation. As mentioned, alterations in Cavβ subunits have been linked to various diseases and pathophysiological disorders, including Brugada syndrome, cardiac hypertrophy, epilepsy, skeletal muscle weakness and high insulin secretion (Antzelevitch et al., 2007; Berggren et al., 2004; Cordeiro et al., 2009; Escayg et al., 2000; Klassen et al., 2011; Ohmori et al., 2008; Pickel et al., 2021; Taylor et al., 2014). Understanding whether nuclear Cavβ2e contributes to pathophysiological mechanisms would be an important direction for future research.

## Data Availability

The datasets presented in this study can be found in online repositories. The names of the repository/repositories and accession number(s) can be found below: https://www.ebi.ac.uk/pride/, accession number PXD058727.
